# Supplementary material for: Redox Potential and Crystal Chemistry of Hexanuclear Cluster Compounds
Source: Molecules. 2021 May 21;26(11):3069. doi: 10.3390/molecules26113069 (PMC8196692; doi:10.3390/molecules26113069)
Supplement: Supplementary file 1 [file molecules-26-03069-s001.zip › molecules-1217326-supplementary.pdf]

Supporting Information

Redox Potential and Crystal Chemistry of Hexanuclear Cluster Compounds

Elena Levi,[a*] Doron Aurbach[a] and Carlo Gatti[b,c*]

Table S1. Average TM-TM distances and the results of the BVS calculations for the TM_6-cluster compounds used in Figure 5

Compound	Average TM-TM distance, Å	BVS_{TM-TM}, v.u.	TM ionic charge, v.u.	Crystal structure ref.
$[Re_6S_8(CH_3NC)_6](CF_3SO_3)_2$	2.603	2.642	4.358	[1]
$[Re_6Se_8(CH_3NC)_6](BF_4)_2$	2.633	2.355	4.645	
$[Re_6Te_8(CH_3NC)_6](BF_4)_2$	2.688	1.904	5.096	
$[Ph_4P]_4[Re_6S_8(CN)_6]*(CH_3CN)_2$	2.609	2.585	4.415	[2]
$Cs_3Na[Re_6S_8(CN)_6]$	2.602	2.653	4.347	[3]
$(EDT\text{-}TTF\text{-}CONH_2)_6[Re_6Se_8(CN)_6]$	2.640	2.295	4.705	[4]
$[NMe_4]_4[Re_6Se_8(CN)_6]*3^1/_3\ H_2O$	2.633	2.352	4.648	[5]
$[Mn(salen)]_4[Re_6Te_8(CN)_6]$	2.693	1.871	5.129	[6]
$[Cu(dien)]_2[Re_6Te_8(CN)_6]$	2.692	1.879	5.121	[7]

Table S1. Continuation

Compound	Average TM-TM distance, Å	BVS $_{TM-TM}$, v.u.	TM ionic charge, v.u.	Crystal structure ref.
$(Bu_4N)_2Mo_6Cl_8Cl_6$	2.603	3.047	2.953	[8]
$Cu_2Mo_6Cl_8Cl_6$	2.605	3.025	2.975	[9]
$Cu_2Mo_6Br_8Br_6$	2.628	2.823	3.177	
$Cs_2Mo_6Br_8Br_6$	2.635	2.770	3.230	[10]
$Cs_2Mo_6I_8I_6$	2.678	2.438	3.562	
$Cu_2Mo_6I_8I_6$	2.672	2.487	3.513	[9]
$(Bu_4N)_2Mo_6I_8I_6$	2.683	2.404	3.596	[11]
$(Bu_4N)_2Mo_6Cl_8Br_6$	2.604	3.038	2.962	[8]
$(Bu_4N)_2Mo_6Br_8Cl_6$	2.630	2.813	3.187	[12]
$(Bu_4N)_2[Mo_6Br_8](CF_3COO)_6$	2.622	2.874	3.126	[13]
$(Bu_4N)_2[Mo_6I_8](CF_3COO)_6$	2.664	2.539	3.461	
$(TBA)_2[Mo_6Cl_7S]Cl_6$	2.626	2.847	3.153	[14]
$(TBA)_2[Mo_6Cl_7Se]Cl_6$	2.629	2.817	3.183	
$(TBA)_2[Mo_6Br_7S]Cl_6$	2.652	2.636	3.364	

Table S1. Continuation

Compound	Average TM-TM distance, Å	BVS_{TM-TM}, v.u.	TM ionic charge, v.u.	Crystal structure ref.
$(Bu_4N)_2W_6Cl_8Cl_6$	2.607	3.124	2.876	[15]
$(Bu_4N)_2W_6Br_8Br_6$	2.635	2.834	3.166	
Cubic-$Cu_2Mo_6Br_8Br_6$	2.631	2.873	3.127	[16]
Orthorombic-$Cu_2Mo_6Br_8Br_6$	2.629	2.897	3.103	
$K_2W_6Br_8Br_6$	2.632	2.858	3.142	
$Ag_2W_6Br_8Br_6$	2.633	2.856	3.144	[17]
$(Bu_4N)_2W_6I_8I_6$	2.671	2.504	3.496	[15]

1. Mikhaylov, M.A.; Mironova, A.D.; Brylev, K.A.; Sukhikh, T.S.; Eltsov, I. V; Stass, D. V; Gushchin, A.L.; Kitamura, N.; Sokolov, M.N. Functionalization of [Re6Q8(CN)6]4− clusters by methylation of cyanide ligands. *New J. Chem.* **2019**, *43*, 16338–16348, doi:10.1039/C9NJ02971K.

2. Baudron, S.A.; Deluzet, A.; Boubekeur, K.; Batail, P. Jahn–Teller distortion of the open-shell 23-electron chalcogenide rhenium cluster cores in crystals of the series, {[Re6Q8]3+(X−)6}3− (Q = S, Se; X = Cl, CN). *Chem. Commun.* **2002**, 2124–2125, doi:10.1039/B205241E.

3. Beauvais, L.G.; Shores, M.P.; Long, J.R. Cyano-Bridged Re6Q8 (Q = S, Se) Cluster-Metal Framework Solids: A New Class of Porous Materials. *Chem. Mater.* **1998**, *10*, 3783–3786, doi:10.1021/cm980564q.

4. Baudron, S.A.; Batail, P.; Coulon, C.; Clérac, R.; Canadell, E.; Laukhin, V.; Melzi, R.; Wzietek, P.; Jérome, D.; Auban-Senzier, P.; et al. (EDT-TTF-CONH2)6[Re6Se8(CN)6], a Metallic Kagome-Type Organic−Inorganic Hybrid Compound: Electronic Instability, Molecular Motion, and Charge Localization. *J. Am. Chem. Soc.* **2005**, *127*, 11785–11797, doi:10.1021/ja0523385.

5. Mironov, Y. V; Cody, J.A.; Albrecht-Schmitt, T.E.; Ibers, J.A. Cocrystallized Mixtures and Multiple Geometries: Syntheses, Structures, and NMR Spectroscopy of the Re6 Clusters [NMe4]4[Re6(Te8-nSen)(CN)6] (n = 0−8). *J. Am. Chem. Soc.* **1997**, *119*, 493–498, doi:10.1021/ja962264k.

6. Kim, Y.; Park, S.-M.; Nam, W.; Kim, S.-J. Crystal structure of the two-dimensional framework [Mn(salen)][ReTe(CN)] [salen = ′-ethylenebis(salicylideneaminato)]. *Chem. Commun.* **2001**, 1470–1471, doi:10.1039/B104276A.

7. Brylev, K.A.; Naumov, N.G.; Fedorov, V.E.; Ibers, J.A. New complex compounds based on [Re6Te8(CN)6]4− cluster anions and [M(dien)2]2+ (M = Co2+ and Cu2+) cations: Adjustment of the crystal structure by the blocking of coordination sites. *J. Struct. Chem.* **2005**, *46*, S130–S136, doi:10.1007/s10947-006-0163-5.

8. Preetz, W.; Harder, K.; von Schnering, H.G.; Kliche, G.; Peters, K. Synthesis, structure

and properties of the cluster anions [(Mo6Cl8i)X6a]2− with Xa=F, Cl, Br, I. *J. Alloys Compd.* **1992**, *183*, 413–429, doi:https://doi.org/10.1016/0925-8388(92)90763-Y.

9. Peppenhorst, A.; Keller, H.-L. Trigonal-planare CuX3-Gruppen in Cu2Mo6X14, X = Cl, Br, I. *Zeitschrift für Anorg. und Allg. Chemie* **1996**, *622*, 663–669, doi:https://doi.org/10.1002/zaac.19966220415.

10. Kirakci, K.; Cordier, S.; Perrin, C. Synthesis and Characterization of Cs2Mo6X14 (X = Br or I) Hexamolybdenum Cluster Halides: Efficient Mo6 Cluster Precursors for Solution Chemistry Syntheses. *Zeitschrift für Anorg. und Allg. Chemie* **2005**, *631*, 411–416, doi:https://doi.org/10.1002/zaac.200400281.

11. Brückner, P.; Preetz, W.; Pünjer, M. Darstellung, Kristallstrukturen, NMR-, Schwingungsspektren und Normalkoordinatenanalyse der Clusteranionen [(Mo6I)Y]2−, Ya = F, Cl, Br, I. *Zeitschrift für Anorg. und Allg. Chemie* **1997**, *623*, 8–17, doi:https://doi.org/10.1002/zaac.19976230103.

12. Preetz, W.; Bublitz, D.; Von Schnering, H.G.; Saßmannshausen, J. Darstellung, Kristallstruktur und spektroskopische Eigenschaften der Clusteranionen [(Mo6Br)X]2− mit Xa = F, Cl, Br, I. *Zeitschrift für Anorg. und Allg. Chemie* **1994**, *620*, 234–246, doi:https://doi.org/10.1002/zaac.19946200207.

13. Kirakci, K.; Kubát, P.; Dušek, M.; Fejfarová, K.; Šícha, V.; Mosinger, J.; Lang, K. A Highly Luminescent Hexanuclear Molybdenum Cluster – A Promising Candidate toward Photoactive Materials. *Eur. J. Inorg. Chem.* **2012**, *2012*, 3107–3111, doi:https://doi.org/10.1002/ejic.201200402.

14. Ebihara, M.; Isobe, K.; Sasaki, Y.; Saito, K. Synthesis, structure, and properties of oxidized hexamolybdenum clusters [(Mo6X7Y)X’6]2- (X = X’ = Cl, Br; Y = S, Se). *Inorg. Chem.* **1992**, *31*, 1644–1649, doi:10.1021/ic00035a024.

15. Zietlow, T.C.; Schaefer, W.P.; Sadeghi, B.; Hua, N.; Gray, H.B. Hexanuclear tungsten cluster structures: tetradecachlorohexatungstate(2-), tetradecabromohexatungstate(2-), and tetradecaiodohexatungstate(2-) relevance to unusual emissive behavior. *Inorg. Chem.* **1986**, *25*, 2195–2198, doi:10.1021/ic00233a019.

16. Zheng, Y.-Q.; Grin, Y.; Peters, K.; von Schnering, H.G. Two Modifications of Copper(I) Octahedro-Hexatungsten(II) Tetradecabromide, Cu2[W6Br14]. *Zeitschrift für Anorg. und Allg. Chemie* **1998**, *624*, 959–964, doi:https://doi.org/10.1002/(SICI)1521-3749(199806)624:6<959::AID-ZAAC959>3.0.CO;2-U.

17. Zheng, Y.-Q.; Borrmann, H.; Grin, Y.; Peters, K.; Schnering, H.G. von The Cluster Compounds Ag[W6Br14] and Ag2[W6Br14]. *Zeitschrift für Anorg. und Allg. Chemie* **1999**, *625*, 2115–2119, doi:https://doi.org/10.1002/(SICI)1521-3749(199912)625:12<2115::AID-ZAAC2115>3.0.CO;2-#.
